# An Exercise Training and Healthy Eating Group Program (ATHENA) for Overweight and Obese Women with Urinary Incontinence: An Intervention Description

**DOI:** 10.3390/healthcare8040575

**Published:** 2020-12-18

**Authors:** Zara Howard, Lynda Ross, Leanne Smith, Nadine Baker, Jennifer Nucifora, Heidi Townsend, Kelly Weir, Shelley Roberts

**Affiliations:** 1 Gold Coast Hospital and Health Service, Southport QLD 4215, Australia; Leanne.Smith6@health.qld.gov.au (L.S.); Nadine.baker@health.qld.gov.au (N.B.); Jennifer.Nucifora@health.qld.gov.au (J.N.); kelly.weir@griffith.edu.au (K.W.); s.roberts@griffith.edu.au (S.R.); 2 School of Exercise and Nutrition Services, Kelvin Grove Campus, Queensland University of Technology, Kelvin Grove QLD 4059, Australia; l20.ross@qut.edu.au; 3 School of Allied Health Sciences, Gold Coast Campus, Griffith University, Southport QLD 4215, Australia; 4 Consumer Researcher, Gold Coast QLD 4215, Australia; hidsta@gmail.com; 5 Menzies Health Institute Queensland, Gold Coast Campus, Griffith University, Southport QLD 4215, Australia

**Keywords:** urinary incontinence, physical activity, nutrition, pelvic floor muscle training, group education

## Abstract

Background: Despite strong evidence for supervised pelvic floor muscle training (PFMT) for women with urinary incontinence (UI), and weight loss and exercise for overweight and obese women with UI, implementation literature on these combined interventions is limited. This paper aimed to describe the rigorous and systematic processes involved in the collaborative development, implementation, refinement and evaluation of a novel, holistic 12 week exercise training and healthy eating group program (ATHENA) for overweight and obese women with UI. Methods/Design: This intervention description paper is part of a larger mixed-methods feasibility study of implementing the ATHENA intervention within a physiotherapy service at a public hospital in Australia. The collaborative intervention design had input from clinicians, researchers and a consumer representative. Results: The intervention involved four evidence-based components—(1) supervised PFMT; (2) general exercise training; (3) pelvic health education; and (4) healthy eating education—delivered face to face over a 12 week period. Supporting resources developed included a Facilitator’s Guide and Participant Workbook. Conclusion: ATHENA is an evidence-based, multifaceted, group-based intervention targeting exercise training and healthy eating for management of UI for overweight and obese women. The structured development process and transparency of intervention content and resources aims to enhance practical application and success in future studies.

## 1. Introduction

Urinary incontinence (UI) is the occurrence of involuntary loss of urine [1]. It affects up to 25% of women aged 14–21 years [2]; 30–57% of middle-aged and post-menopausal women [3]; and 75% of elderly women aged ≥75 [4,5]. UI has profound effects on quality of life, physical and psychological well-being, and socio-economic aspects of a woman’s life [6]. It also places a large economic burden on health care systems [5,7]; in Australia, the estimated cost of UI treatment was projected to be AU$1.27 billion in 2018 [8]. The pathophysiology of UI is often multifactorial and includes pelvic floor muscle dysfunction, parity, ageing and lifestyle-related factors such as obesity [9]. Despite the large burden that UI places on women, many avoid or delay seeking treatment, usually due to embarrassment or shame [10,11,12].

Evidence-based clinical practice guidelines for the conservative management of UI recommend intensive, supervised pelvic floor muscle training (PFMT) as first-line treatment for women with UI [7,13]. This is supported by high-quality evidence showing that PFMT increases continence and improves quality of life among this group [7]. The guidelines also recommend weight management for overweight and obese women with UI, as overweight is associated with a one-third increase in UI risk and obesity doubles the risk [7,14,15]. Overweight and obesity are thought to contribute to UI through chronically increased intra-abdominal pressure, caused by excess weight, bearing down on the bladder and pelvic tissues [15]. A significant proportion of women living with UI are overweight or obese [15,16]; and the recommended management of both conditions includes exercise (PFMT for UI [7]; and general exercise for weight loss in overweight/obesity [17]). However, UI and high weight are both barriers to exercise [18]. It therefore seems appropriate and efficient to develop interventions that target both issues (given the overlapping guideline recommendations), while taking into account the barriers each condition brings. Yet, despite high-quality evidence for intensive, supervised PFMT and weight management strategies, previous studies have tested these interventions individually and have not used a combined approach [19,20].

It is challenging for Australian health services to provide the level of care stipulated in clinical practice guidelines to patients with UI. This may largely be due to an ageing population, high rates of overweight and obesity, and public health services operating at capacity with increasing numbers of UI referrals. An audit of our hospital’s urogynaecology waitlist found a significant increase (139%) in new referrals per month from 2016 to 2017, highlighting the increasing demand for continence service provision. Furthermore, an audit of the Women’s Health Physiotherapy (WHP) service identified that over half of new patients with pelvic floor conditions were overweight or obese. Usual care for these patients consisted of one-on-one consultations with physiotherapists and the prescription of a home-based PFMT program, but did not include the combined weight management and PFMT approach, or the frequency of supervised PFMT recommended by evidenced-based clinical practice guidelines, thus highlighting a clear evidence–practice gap. In order to deliver care conforming with evidence-based guidelines, an innovative approach that was feasible, effective, and sustainable to be integrated into the WHP service within existing resources was required. As stated earlier, a multicomponent intervention, incorporating several of the strategies recommended by guidelines, may be an efficient and effective way to manage UI among overweight and obese women, who make up the majority of patients presenting to the WHP service. Furthermore, a group-based program may promote behaviour modification and increase adherence to treatment, through provision of mutual support, information sharing, and peer motivation [21]. It may also be an efficient way to meet the increasing demand of continence service provision.

As such, our team has developed a novel, multifaceted, group-based lifestyle intervention with both physiotherapy and dietary components—an exercise training and healthy eating group program (ATHENA), for overweight/obese women with UI—in order to manage the increasing demand for continence service provision. ATHENA is the product of translating evidence-based clinical practice guidelines for UI into usual practice in a feasible, effective and sustainable way, within the resources and budget of the health service. Given that research translation is a complex process, we are providing this detailed description of the ATHENA program’s development to give a real-world example of how this process can occur in practice, with limited resources. This brings important insights to the field of knowledge translation (KT) and to health services research more broadly, given behaviour change interventions are only described in detail 5–30% of the time and that adequate description of interventions is key for their further development and scale up [22]. Thorough description of intervention development also allows for adequate understanding, replicability and evaluation of KT interventions [22], which is vital for advancing the science. We used an integrated KT approach when designing ATHENA, in which knowledge end users were engaged throughout the research process, an approach considered to result in interventions that are more likely to be relevant, acceptable and useful to end users [23]. As the reporting of integrated KT approaches is lacking in the literature (particularly among group-based lifestyle interventions) and studies that do report on integrated KT do so poorly, this paper also brings further insights on the research co-design process to the literature.

This holistic, group-based intervention may be effective in promoting behavioural exercise and dietary changes for weight loss whilst providing regular, supervised PFMT for overweight and obese women with UI. It may also be an efficient way to deliver the care recommended by clinical practice guidelines, by using a group-based approach [21,24]. While we will report on the effects of ATHENA (on UI and weight) and its acceptability to patients separately, this paper provides a detailed outline of how the intervention was co-developed (with knowledge users), implemented (in usual practice), and refined. We have also provided the knowledge tools (intervention materials) that were produced as Supplementary Material, so that others can access, adapt and use these in their own settings, a key step in knowledge translation.

## 2. Materials and Methods

### 2.1. Overview

The current study describes the rigorous and systematic processes undertaken to collaboratively develop, implement, refine, and evaluate the ATHENA intervention for overweight and obese women with UI. This paper is part of a mixed-methods feasibility study that was conducted within the WHP service at a large, public, tertiary hospital in Queensland, Australia. This study was approved by relevant hospital (reference number: HREC/2018/QGC/46582) and university (GU reference number: 2018/960; QUT reference number: 1900000961) Human Research Ethics Committees.

### 2.2. Procedure

**Figure 1:**
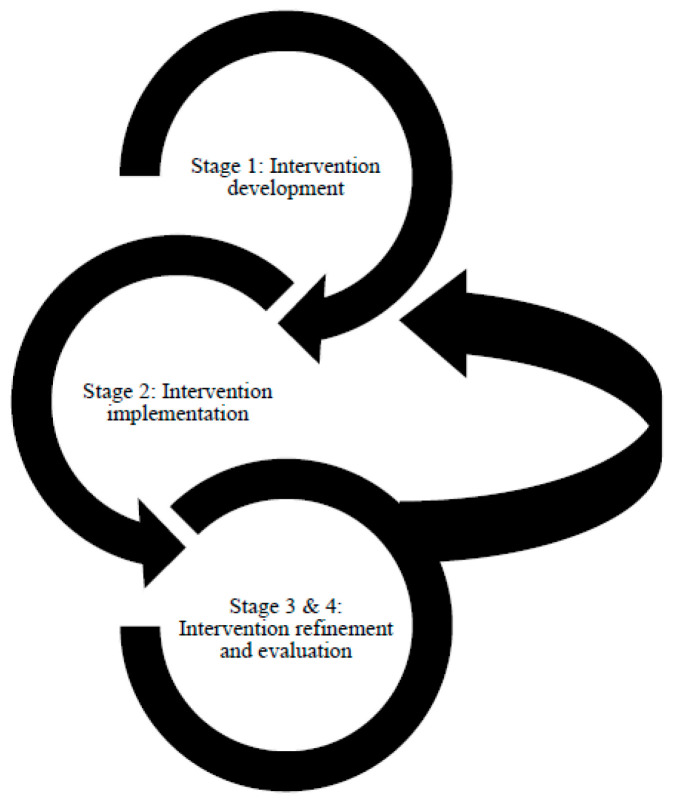
Processes guiding the implementation of the ATHENA intervention.

The development of the ATHENA intervention required an innovative approach to translate evidence-based UI guidelines into routine clinical practice. An integrated knowledge translation (research co-production) [23] approach was used, guided by Graham’s Knowledge to Action process [25]. The collaborative development, implementation, refinement and evaluation of the ATHENA intervention is outlined below, in four stages (see Figure 1).

#### 2.2.1. Stage 1: Intervention Development

The ATHENA intervention and supporting clinician and participant resources were developed by the research co-production team; and human resources, equipment and consumables were secured via consultation with and support from key stakeholders (Director of Physiotherapy, Assistant Director of Physiotherapy (Women’s Health and Cancer Care), Assistant Director of Physiotherapy (Outpatients) and Allied Health Research Team) over a three-month period in October–December 2018.

##### Establishment of Research Co-Production Team

A multidisciplinary research team was formed in October 2018, including physiotherapists, dietitians, allied health research fellows and a health consumer. This team worked together on all aspects of intervention development, implementation and refinement. Three skilled physiotherapists (JN, LS, ZH) with extensive clinical experience and training in continence and women’s health, who were appropriately positioned in the WHP service, designed recruitment procedures and intervention strategies for women with UI (note for readability, these continence and women’s health physiotherapists will be referred to as physiotherapists for remainder of the paper). Three dietitians were also members of the research team, including a clinical dietitian experienced in nutrition education and weight loss who practiced in the physiotherapy outpatient clinic (NB); and two academic dietitians, one experienced in group-based weight loss interventions (LR); and one allied health research fellow with consumer engagement and implementation research experience (SR). A second allied health research fellow with a background in speech pathology and research co-production who had previous experience in research in the WHP service (KW) and a health consumer who had personal experience with both UI and the WHP service (HT) were also part of the research co-production team.

##### Collaborative Design of Evidence-Based Intervention and Supporting Clinician and Patient Resources

Evidence-based guidelines recommend intensive, supervised PFMT for 8–12 weeks [26]; and weight loss for overweight and obese women via lifestyle interventions that target dietary and exercise behaviour change [17] as first-line management for UI. Group-based treatments may enhance patient motivation through peer support and be a cost-effective mode of service delivery [21]. Further, multicomponent interventions delivered using multidisciplinary care may be more effective than those delivered by individual health professionals [17]. As such, ATHENA was developed as a weekly, face-to-face, group-based intervention run over a 12 week period, comprising four evidence-based components: (1) PFMT^a^; (2) general exercise training (for weight loss)^b^; (3) pelvic health education^c^; and (4) healthy eating education^b^ (^a^strong recommendation, high-quality evidence; ^b^strong recommendation, moderate quality evidence; ^c^moderate-quality evidence).

The exercise component of the ATHENA intervention (PFMT and general exercise training) was held in the physiotherapy outpatient gym for one hour on a weekly basis. The research physiotherapists selected a range of general exercises in the format of high-intensity interval training, with the aim to increase confidence and meaningful participation in physical activity during the ATHENA intervention and for practice at home. The supervised PFMT component was based on training principles of muscle physiology, with functional training incorporating PFMT with increases in intrabdominal pressure and general exercise.

The healthy eating and pelvic health education components were delivered in a private room near the gym for an hour immediately following the PFMT and general exercise training. The healthy eating education was adapted from the evidence-based Healthy Eating and Lifestyle Program (HELP) [27], which focused on dietary and lifestyle modifications and behaviour change techniques to promote weight loss in overweight and obese patients. HELP was developed by Queensland Health dietitians and psychologists to align with the National Health and Medical Research Council [17] and National Institute for Health and Care Excellence guidelines for obesity management [28]. The study team of dietitians (including one experienced in using HELP both in practice and research), physiotherapists and consumer researcher adapted HELP resources for use in ATHENA healthy eating education sessions. The pelvic health education was adapted from previous education provided by physiotherapists within the WHP service and a previous study using a pelvic health group-based education intervention [29].

All aspects of the intervention were delivered by clinicians via open discussion, guided by learning outcomes outlined in the Facilitator’s Manual (see Supplementary Material S1) and reflective and planning tasks in the Participant Workbook (see Supplementary Material S2). This ensured key content was covered and allowed participants to share personal experiences relating to each topic, reflect on barriers to behaviour change, and devise strategies to overcome these barriers. The Participant Workbook was developed with careful consideration of educational content and language using analogies, alliterations and rhyming to assist consumer learning. The Participant Workbook was tested for readability in Microsoft Word using the Flesch Reading Ease (score 0–100, with a high score being easier to read), which scored 68.9, and Flesch–Kincaid Grade level (based on United States school grade level), which scored 6.0. At the end of each weekly ATHENA session, participants were encouraged to write down take-home messages and fill out an action plan for the following week, to be reviewed at their next ATHENA session.

##### Resources

Physical resources required to deliver the intervention included (1) infrastructure: gym space with rails, chairs, towels, surface cleaning and first aid equipment, water fountain, and music; and a private room with whiteboard, chairs and table; (2) equipment: pelvic anatomy models and diagrams, food consumption models, and exercise equipment (theraband, hand held weights, floor mats, steps, fitness balls, and bike); and (3) consumables: stationery, printouts of Participant Workbooks and other resources (e.g., exercise handouts). To implement the intervention, the following human resources were required: ATHENA coordinator (1 h/week); physiotherapist (2 h/week); dietitian (1 h/week); and administrative officer (1 h/week).

##### Establishment of Intervention Team

The establishment of the intervention team was finalised in December 2018. The ATHENA coordinator ensured that all administrative processes were completed correctly (i.e., participant scheduling, gym and room bookings) and that clinicians delivering weekly ATHENA sessions were orientated and organised. Lead researcher, ZH, was the ATHENA coordinator for the first four months (January–April 2019), followed by LS for the remaining months (May–December 2019). Physiotherapists on the research team (JN, LS, ZH) and in WHP service recruited participants and delivered the physiotherapy intervention components: PFMT, general exercise training and pelvic health education (LS, ZH). The dietitian working in the physiotherapy outpatient clinic (NB) was seconded one hour/week to the WHP service to deliver the dietetic component. Leave cover was provided by the academic dietitians (LR, SR) for dietetic components, and by physiotherapists working in the WHP service for physiotherapy components. Consistency in delivering physiotherapy and dietetic education sessions was achieved through development of, and training on, the Facilitator’s Guide (Supplementary Material S1). All clinicians who delivered ATHENA education sessions were orientated to resources, equipment and processes. Administrative staff managed scheduling of participants and printed resources as required. A physiotherapy assistant (1 h/week) would be a useful resource to support the physiotherapist while delivering the exercise component. However, this was unable to be provided secondary to clinical work demands during this study.

#### 2.2.2. Stage 2: Intervention Implementation

The ATHENA intervention was implemented in the existing WHP service in January 2019 at the study hospital. Intervention sessions ran weekly (Tuesday 10 am–12 pm) during the study period (January–December 2019).

##### Participant Recruitment Procedures

Participants were recruited consecutively from January to October 2019 from the WHP service. Women accessing the hospital’s WHP service who were aged ≥18 years, diagnosed with UI and considered overweight or obese (i.e., body mass index ≥25 kg/m^2^) were eligible to participate. Exclusion criteria included contra-indications for exercise (e.g., acute illness, uncontrolled cardiovascular conditions), pregnancy, <3 months post-natal, and being unable to provide informed consent (e.g., poor cognition or non-English speaking background). Physiotherapists screened patients for eligibility within their usual WHP clinics. Patients who met eligibility criteria were given a brief explanation of this study and asked for verbal consent for the research team to contact them. Those who agreed to be contacted were provided with the Participant Information and Consent Form and the ATHENA Promotional Brochure. One week later, an ATHENA coordinator (LS, ZH) phoned potential participants to provide more information about the ATHENA study and intervention, and to answer any questions. Patients who chose to participate were enrolled in this study and booked into an ATHENA session, to which they were instructed to bring their signed consent form.

##### Participant Experience

Participants were invited to attend ATHENA sessions over a 12 week period. The pelvic health and healthy eating education components included four topics that ran consecutively over four weeks (rolling in nature; minimum attendance required all education sessions to be completed). If participants missed an education topic, they could attend the next one offered in four weeks’ time; and were also able to repeat education topics if they wished. The supervised PFMT and general exercise training ran on a weekly basis for the entire 12 weeks (minimum attendance: four weeks of exercise training components). There were no more than ten participants at each ATHENA session to allow for adequate supervision of PFMT and general exercise training.

#### 2.2.3. Stage 3: Intervention Refinement

Fortnightly debriefing and evaluation of intervention delivery processes were completed during the period January–February 2019 by members of the research co-production team, with most feedback coming from the consumer co-investigator (HT), who had attended some ATHENA sessions. This resulted in minor revisions to intervention resources and delivery processes to ensure they met participants’ needs. After the initial eight weeks of the intervention implementation there were no further changes to allow consistency in intervention delivery.

#### 2.2.4. Stage 4: Intervention Evaluation

The intervention was implemented and evaluated concurrently using a hybrid implementation-effectiveness design [30], which allows for rapid translation of research (in this case, evidence-based guideline recommendations) into practice. Implementation processes, including reach, dose and fidelity/adaptations, and intervention acceptability and feasibility, were assessed via process evaluation. At the same time, ATHENA’s clinical effectiveness was evaluated using pre-/post-measures of UI symptoms and quality of life. These results will be published in a future article.

## 3. Results

### 3.1. Intervention Description

The ATHENA intervention consisted of four components, as described in detail below. For successful implementation, these four components were supported by the Facilitator’s Guide (Supplementary Material S1) and Participant Workbook (Supplementary Material S2).

#### 3.1.1. Component 1: Supervised PFMT

The first component of ATHENA aimed to meet evidence-based clinical practice guidelines [7] that recommend weekly, supervised PFMT for treatment of UI. Prior to attending the group intervention, participants were assessed for correct PFM contraction technique at their one-on-one physiotherapy appointment and were provided with an individualised PFMT program to complete as part of a home exercise program. This aimed to increase participants’ confidence in correctly activating pelvic floor muscles during the supervised PFMT component of the ATHENA intervention, which ran for 10–20 min at the physiotherapy outpatient gym during the warm up and cool down of the general exercise training session. It included PFM contractions of different intensity, duration, and a functional PFMT component during the general exercise training session and activities that increase intra-abdominal pressure [31,32]. Please refer to the Facilitator’s Guide (Supplementary Material S1) for full details.

#### 3.1.2. Component 2: General Exercise Training

The second component of the intervention aimed to increase participation in meaningful physical activity to assist with weight loss as recommended by clinical practice guidelines [7]. The general exercise training component was led by the physiotherapist at the WHP outpatient gym, using existing equipment and resources, and ran for 30 min at each ATHENA session. The general exercise training was formatted with a 5 min warm up; 20 min of high-intensity interval training (HIIT); and 5 min cool down. The HIIT involved three repetitions of 45 s work to a 15 s rest ratio through five stations. Each station targeted a different exercise type: (1) cardio; (2) upper body; (3) lower body; (4) abdominal; and (5) balance and jumping; with three options for levels of difficulty (beginner, intermediate and advanced) for participants to choose from. The exercises changed each week for variety. The cool down was completed on floor mats. Throughout general exercise training, individualised modification of exercise and functional integration of PFMT was guided by physiotherapists. At completion of each training session, participants were given a handout of the exercises they completed that day for independent practice at home. To ensure patient safety, each exercise and use of equipment were demonstrated prior to commencement of the general exercise training session. Consistent with American College of Sports Medicine recommendation for modest weight loss, participants were encouraged to complete 150–250 min/week of moderate intensity cardiovascular-based physical activity (as per Borg’s rate of perceived exertion scale [33]) as part of their home exercise program.

#### 3.1.3. Component 3: Pelvic Health Education

The third component of the intervention aimed to increase participants’ knowledge on different pelvic health topics, through open-forum group education and discussion. This is an integral aspect of the intervention, as knowledge about the function of pelvic floor muscles and their activation has been shown to reduce symptoms of pelvic floor muscle dysfunction and improve quality of life [34]. Pelvic health education sessions were led by a physiotherapist and included four education topics, which ran for 20–25 min over four weeks. Sessions were conducted weekly after each gym session in a private room conveniently located near the gym. Each pelvic health education topic targeted a theme: (1) Powerful Pelvis, (2) Beautifully Behaved Bladder, (3) Terrific No. Two’s and (4) Learning to Link. Full details of these topics, including target learning outcomes, are outlined in the Facilitator’s Guide (Supplementary Material S1).

#### 3.1.4. Component 4: Healthy Eating Education

The fourth and final component of the intervention aimed to improve healthy eating behaviours and complemented the general exercise training component to achieve non-surgical weight loss to reduce UI (as recommended for overweight and obese women [7]). This component was led by dietitians and included four education topics of 40–60 min each that ran over four weeks in a private room conveniently located near the gym. Each session targeted a separate topic: (1) Enjoy Eating, (2) Powerful Portions, (3) Shop ‘til You Drop and (4) Mood and Food. Full details including target learning outcomes are outlined in the Facilitator’s Guide (see Supplementary Material S1). The four topics were targeted to educate and motivate participants to improve their dietary intake in order to facilitate weight loss. Building from the foundation of the Australian Dietary Guidelines and serving recommendations specified in the Australian Guide to Healthy eating, the dietitians encouraged consistent behaviour and lifestyle modifications to enable the establishment of improved nutrition and food choices. For each topic, dietitians provided verbal education and facilitated open discussions aligned with the learning outcomes in the Participant Workbook (see Supplementary Material S2). Participants were also provided with additional handouts, including nutrition label-reading cards to guide food shopping; and were shown food models, portion plates and packaging samples, to enhance their interactive learning experience.

## 4. Discussions

This paper describes the development, implementation, refinement and evaluation of ATHENA, a novel exercise training and healthy eating group program for overweight and obese women with UI. The ATHENA intervention was developed to facilitate the translation of evidence-based clinical practice guidelines [26] into usual practice in a real-world setting. The resultant knowledge products include the 12 week ATHENA program and supporting materials, including a Participant Workbook and Facilitator’s Guide, which we have made available as Supplementary Material to this paper to further support knowledge translation. This paper may assist future research and clinical practice endeavors by providing a comprehensive description of intervention co-development with key stakeholders, its pragmatic implementation in usual practice, and its refinement and evaluation plan.

The success of the ATHENA intervention’s development was largely attributed to the rigorous co-production/integrated KT approach underpinned by high quality, evidence-based clinical practice guidelines. The varied expertise of and collaboration between members of the research team allowed for pragmatic integration of recommendations into practice, within an existing hospital service. The inclusion of both clinician and academic researchers on the study team, who had content expertise in physiotherapy and dietary interventions, and experience in research co-production and KT, was vital to success. The inclusion of highly skilled continence and women’s health physiotherapists who worked within the health service (and hence had a broad understanding of study context) ensured the intervention was relevant to the target population and feasible to deliver within the health service’s existing resources. Having consumer co-investigator (HT) involvement throughout the research process provided a unique ‘insider perspective’ to the consumer experience of intervention content and delivery. This co-production approach is recognized to produce interventions that are likely to be relevant and acceptable to end users [25], potentially increasing uptake and effectiveness [35] and value-adding to researcher and health service perspectives [36]. Early involvement of the consumer researcher (i.e., from the intervention design stage), early identification of team strengths and expertise, regular team meetings, a flexible and pragmatic approach to research design, shared team responsibility and decision making, and provision of research support were strategies used to optimize the intervention’s success [35].

The delivery style adopted was key to participants’ understanding of and involvement in the intervention; and reflected principles of patient participation in care. Patients who take ownership and are actively involved in their own care have been found to have improved health outcomes and higher satisfaction with care [37,38]. Sahlsten et al. outline four concepts of patient participation in care: (1) a mutually trusting and respectful relationship between patient and clinician; (2) surrendering of some power/control by clinicians; (3) meaningful exchange of knowledge/information; and (4) active mutual engagement in intellectual and/or physical health care activities [39]. These concepts were apparent in the ATHENA intervention. For example, guiding principles were outlined to participants at their initial session using the Participant Workbook to foster positive, respectful and connected relationships between clinicians and participants, and between participants themselves, from program commencement. Delivery of education topics in an open forum, allowing participants to ask questions and guide the direction of discussions, required surrendering of control by clinicians and allowed for information exchange between clinicians and participants, and between participants. Finally, facilitators (physiotherapists and dietitians) actively engaged participants in both intellectual and physical activities (education sessions, PFMT/general exercise training) throughout the program for health improvement. Clinicians focused on small, achievable lifestyle changes and provided support and encouragement to participants rather than being prescriptive; and information and activities were tailored to individuals, consistent with a patient-centered care approach.

Educational components of ATHENA also took into consideration participants’ health literacy, another important aspect of patient-centered care. Health literacy is a broad term that encompasses how people access, understand and use health information [40]. People with low health literacy are less likely to access health services, understand care instructions or self-manage their care; and are more likely to have worse overall health outcomes [40]. On the other hand, those with higher health literacy are more likely to be involved in their health care and decision making [40]. For these reasons, and as one in six Australians feel that they are unable to appraise health information [41], educational materials must be developed to cater to patients from a range of backgrounds and literacy levels. The ATHENA Participant Workbook, which contained all educational content, was designed to be easy to read and understand, with a Flesch Reading Ease score of 68.9 and a Flesch–Kincaid Grade level score of 6.0 (6th grade reading level). It also included tables, pictures and figures to reduce text where possible. Importantly, clinicians’ delivery of the educational content aimed to optimize learning, and followed recommendations for effective counselling in urogynecology outlined by Balzarro et al. [42]. Firstly, multiple modes were used to deliver ATHENA educational content, including written and verbal information, interactive group discussions, pictures and diagrams, and food models. Next, it was delivered in a quiet, private and comfortable setting, by calm, non-judgmental and empathetic clinicians, who used simple language and checked patients’ understanding of educational content. Clinicians also encouraged patient involvement in group discussions, so patients could learn not only from clinicians, but from each other. Patients’ ideas and expectations of ATHENA, fears and concerns about their condition, motivation for treatment, and their understanding of educational content were common themes in these discussions, consistent with Balzarro et al.’s guidance [42].

## 5. Conclusions

This intervention description paper provides an example of how to co-develop and implement a multidisciplinary exercise and health eating group intervention for overweight and obese women with UI into an existing health service. While the development process was fairly resource intensive, the resultant intervention may lead to cost savings in the future, through more efficient health service delivery and better patient outcomes (if ATHENA is shown to be effective). For example, Lamb and colleagues identified that the average cost of continence treatment provision was £53.37 per participant for individual treatment and £7.73 per group participant (group size approximately ten women) in the United Kingdom’s public health system. With the increasing prevalence and demand on health services to manage UI and obesity, future cost-effectiveness studies of the ATHENA intervention are of importance.

While we plan to publish our findings on ATHENA’s effectiveness for improving UI and weight among overweight/obese women at our health service, its evaluation in other settings is warranted. Future studies should report on any adaptations made to ATHENA and how implementation occurs, to assist interpretation of findings. Research should also be undertaken to determine the longer-term effects of ATHENA, including whether patients are able to maintain their lifestyle changes. If proven effective, ATHENA could be adopted in other Australian health services and perhaps more broadly, potentially benefiting patients (through improvements in UI, weight and/or quality of life) and health services (through efficient and effective service delivery).

As outlined previously, a clear evidence–practice gap existed for a holistic, multifaceted, group-based intervention for treating UI in overweight/obese women [19,20]. Details on the development and content of complex, multicomponent behaviour change interventions are often lacking, making their replication in other health services difficult [43,44]. This paper provides important insights into knowledge translation by detailing the pragmatic co-development and implementation of a complex behaviour change intervention in a real-world clinical setting. We have also provided the knowledge products (intervention materials) to support ATHENA’s adoption, adaptation and translation into practice, in health services with similar contexts. We encourage others to use these materials, and our experiences (as outlined in this paper), to facilitate or inform their own KT research. Finally, there is also scope for future research on the delivery of ATHENA in community, private practice and/or online settings. This would support wider dissemination and engagement, for example to rural and remote participants who have difficulty accessing health care.

## Supplementary Materials

The following are available online at https://www.mdpi.com/2227-9032/8/4/575/s1, Supplementary Material S1: Facilitator’s Guide; Supplementary Material S2: Participant Workbook.
