# Supplementary material for: An Exercise Training and Healthy Eating Group Program (ATHENA) for Overweight and Obese Women with Urinary Incontinence: An Intervention Description
Source: Healthcare (Basel). 2020 Dec 18;8(4):575. doi: 10.3390/healthcare8040575 (PMC7767144; doi:10.3390/healthcare8040575)
Supplement: Supplementary file 1 [file healthcare-08-00575-s001.zip › Supplementary Material 2.pdf]

Welcome to ATHENA

An Exercise Training and Healthy Eating Group Program

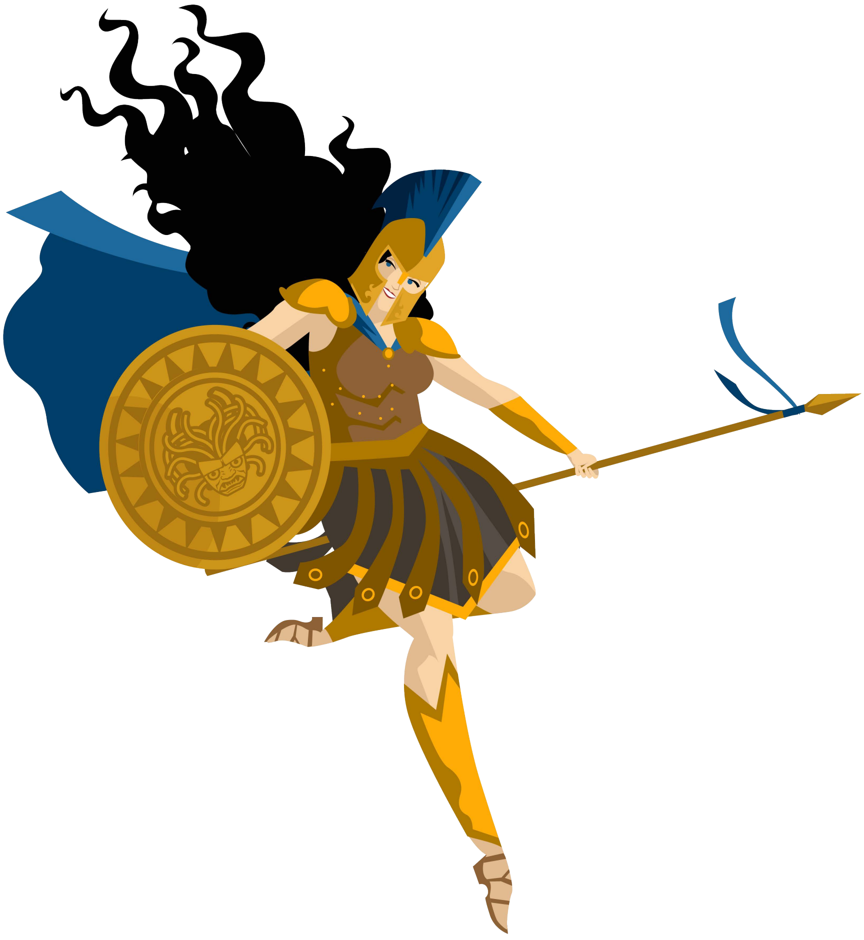

Athena is a greek goddess of Wisdom and War.
She is known for her strength and skill,
and is the patron goddess of heroic endeavour.

Please bring this workbook to each appointment.

Gold Coast Health

Queensland Government

What is ATHENA?

ATHENA is a **12-week** program designed to help you improve your bladder control, manage your weight and improve your overall health.

- The first **4 weeks** are 2 hours with a Physiotherapist and Dietitian
- The following **8 weeks** are 1 hour with a Physiotherapist

You are not alone!

Over 30% of women have bladder control issues, and **1 out of 7** women have these when they exercise. As a result, many women avoid exercise and gain weight, making their bladder issues worse.

You will get great results!

Improving bladder control leads to increased confidence to exercise. This then results in improved weight, and this can improve bladder control further! Other women just like you have had great results from ATHENA:

"I found the program really great. Helpful, informative and a great kick start to a change in lifestyle for me (remembering to do my pelvic floor every day)."

"I really enjoyed the sessions. It really made me focus on my pelvic floor and what food choices I should make."

Letting your inner ATHENA shine:

Have fun! It is okay to laugh, chat and question. We can achieve more together, as a team.

Make it about you: Set personal goals and celebrate these goals with us when you reach them.

Be strong: Strength doesn't just happen in the gym. Strength is important for you as a whole person – in mind, body and spirit.

Lifercise = Life + Exercise: Make exercise fun and a way of life. Completing exercises once a week during the class is not enough to get best results.

Small changes count! Improving your eating habits is a day by day, step by step process. Enjoy noticing the small positive food choices you have made this week compared to last week. They all count!

Recharge the batteries: practice regular sleep hygiene to maximise your energy. Maximised energy = maximized determination!

Resilience: Changes may not happen immediately; be persistent and support each other!

Challenge: Continue to challenge yourself and progress your program. Use your workbook to review progress and plan ahead.

Respect, listen and share: To help make our group environment enjoyable for everyone involved.

Safety: Please make sure you have been shown how to use gym equipment correctly by the Physiotherapist before you attempt yourself.

The 'M' Words: Move More!

Moving more strengthens the pelvic muscles and helps with weight loss!

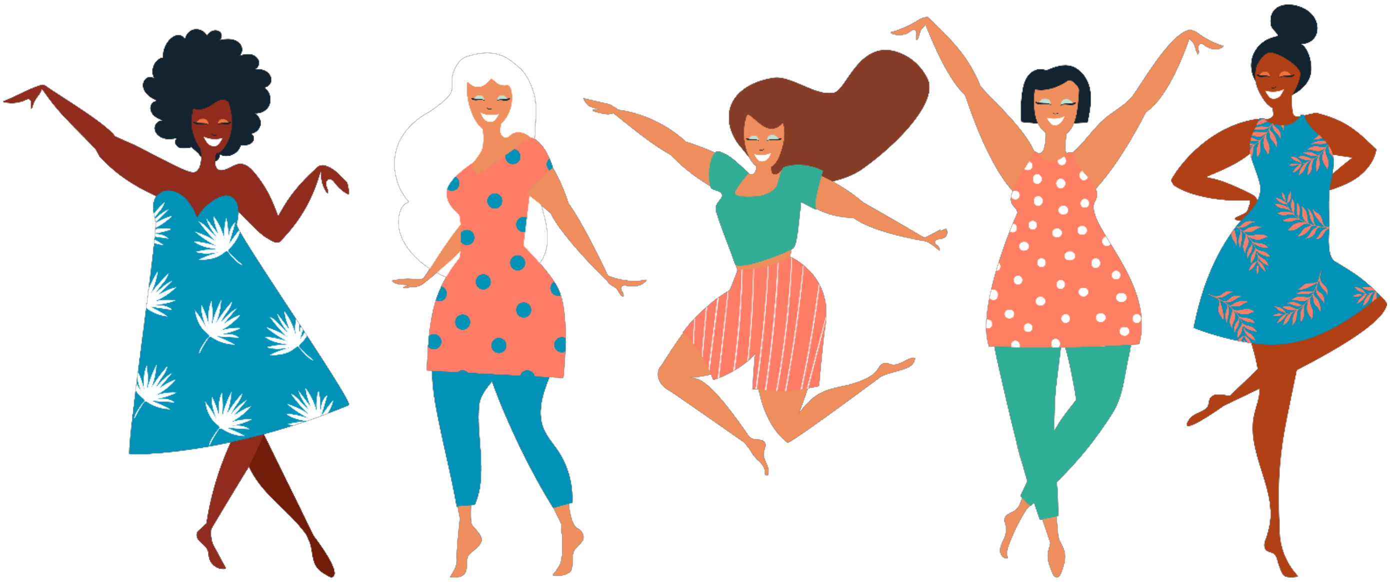

Losing just **5% of body weight** (e.g. for 100kg person, this would be 5kg) can improve bladder control in women who are overweight.

Every day is the way!

A daily dose of 30 minutes of movement at a '*somewhat hard'* to '*hard'* intensity is recommended for weight loss.

Enjoy exercise!

The best exercises are the ones you enjoy. What is rewarding for you, practical, social, accessible, affordable?

General Exercise Notes

Pelvic Muscle (Trampoline) Training

What is the pelvic trampoline?

Just like a trampoline, the pelvic floor muscles can stretch, recoil, are powerful and fun!

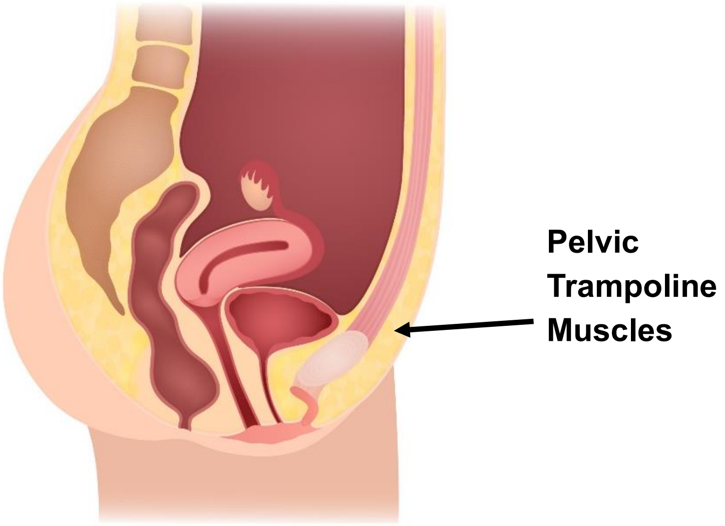

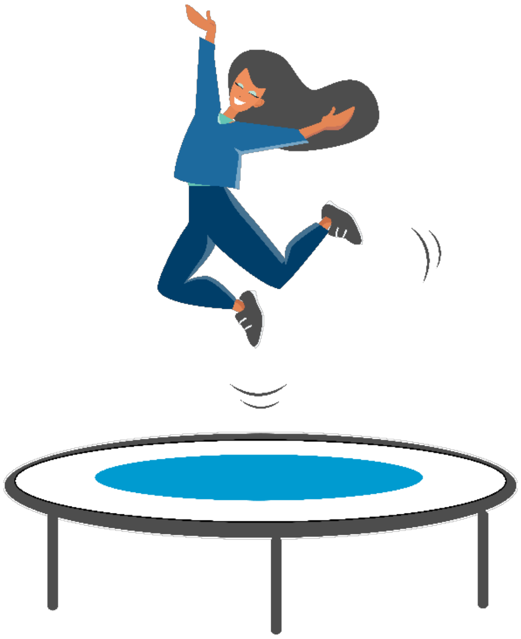

Note: you do <u>NOT</u> need to use a trampoline to exercise these muscles!

Current international guidelines recommend:

- Pelvic muscle exercises should be first-line therapy for women of all ages with stress, urge or mixed urinary leakage.
- Intensive, supervised pelvic muscle exercise training (*e.g. weekly, in the ATHENA group*) achieves better results than just a program at home.

When will I see results?

You may notice some results from completing the pelvic muscle exercises immediately. It takes ***2–3 months*** of daily exercise to strengthen these muscles. This is when you will see the best results.

Pelvic Muscle Exercise Notes

Healthy Pelvic Habits

We know that explaining and exploring healthy pelvic habits not only enriches women's knowledge, but also can also improve bladder control and improve quality of life!

There are 4 key topics that will be covered in healthy pelvic habits:

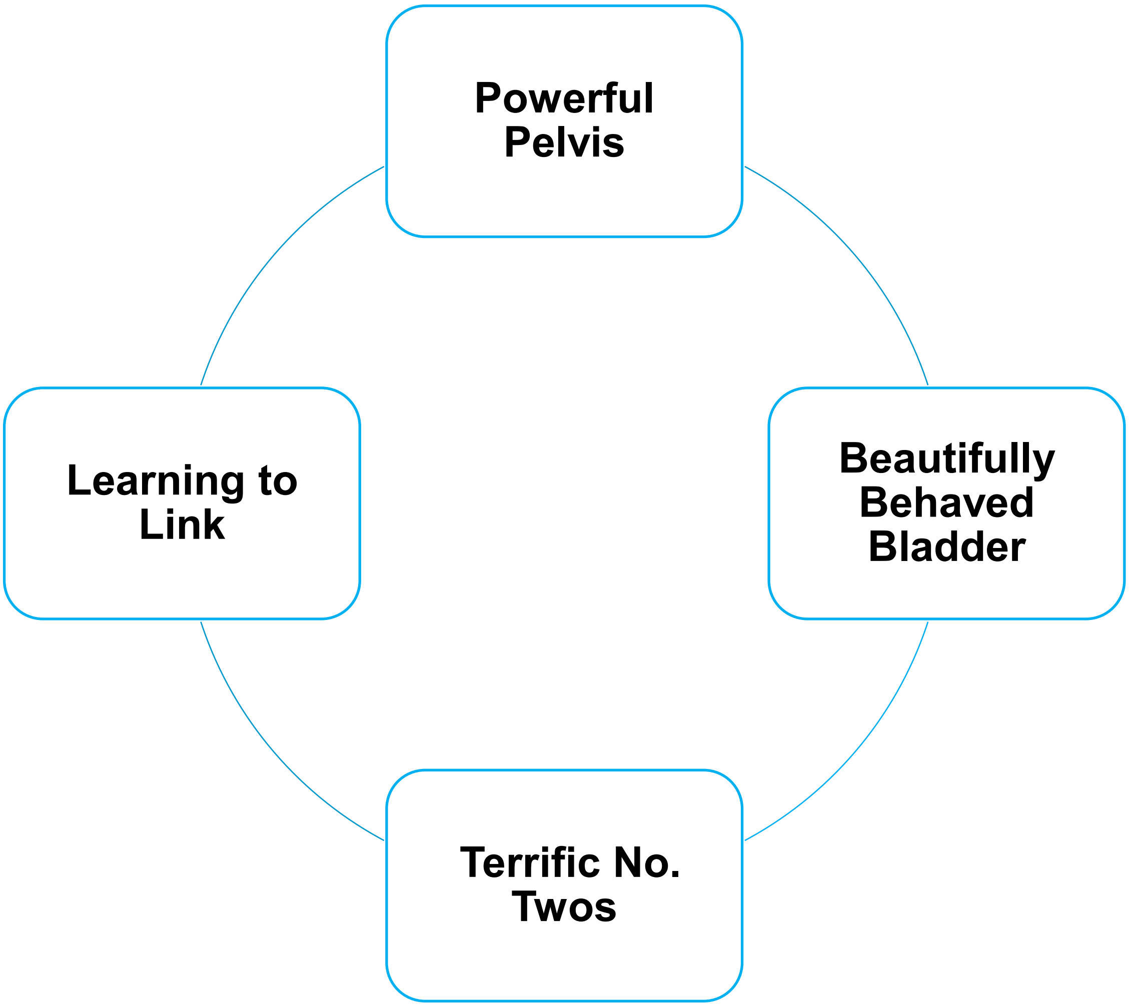

Powerful Pelvis (keeping you strong)

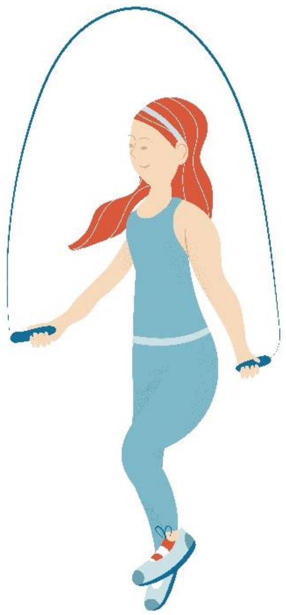

My take home messages

In the next week

I plan to…	I did…

Beautifully Behaved Bladder (keeping you in control)

1.5-2L total fluids / day → Avoid bladder irritants → Delay voiding every 2-3 hours =

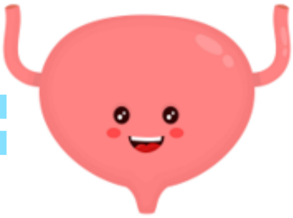

My trigger management plan

What are my triggers?

My trigger management plan for the future

In the next week

I plan to…	I did…

Terrific No. 2's (keeping you regular)

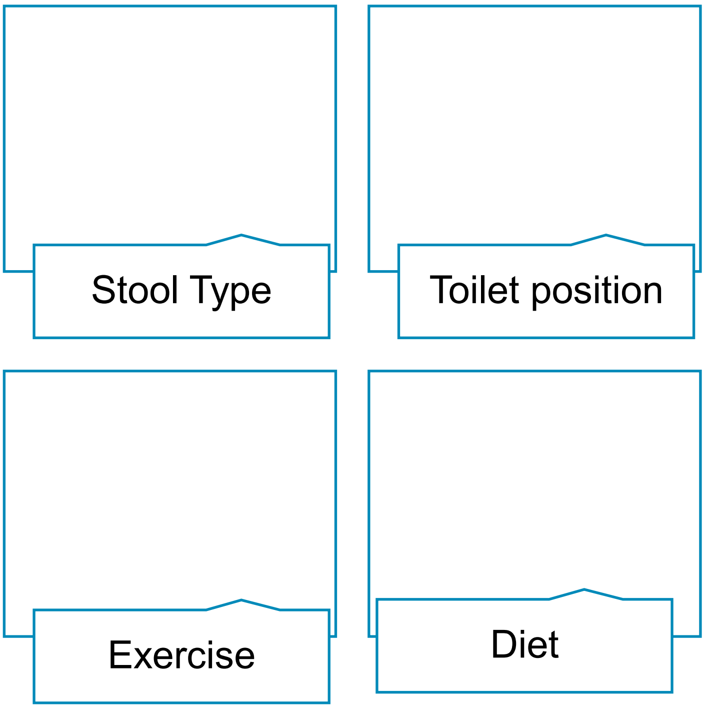

In the next week

**I plan to…**	**I did…**

Notes

Learning to Link (keeping you in tune with your body as a whole)

Bladder	Bowel	Exercise

In the next week

I plan to…	I did…

Notes

Healthy Eating Habits

There are 4 key topics that will be covered:

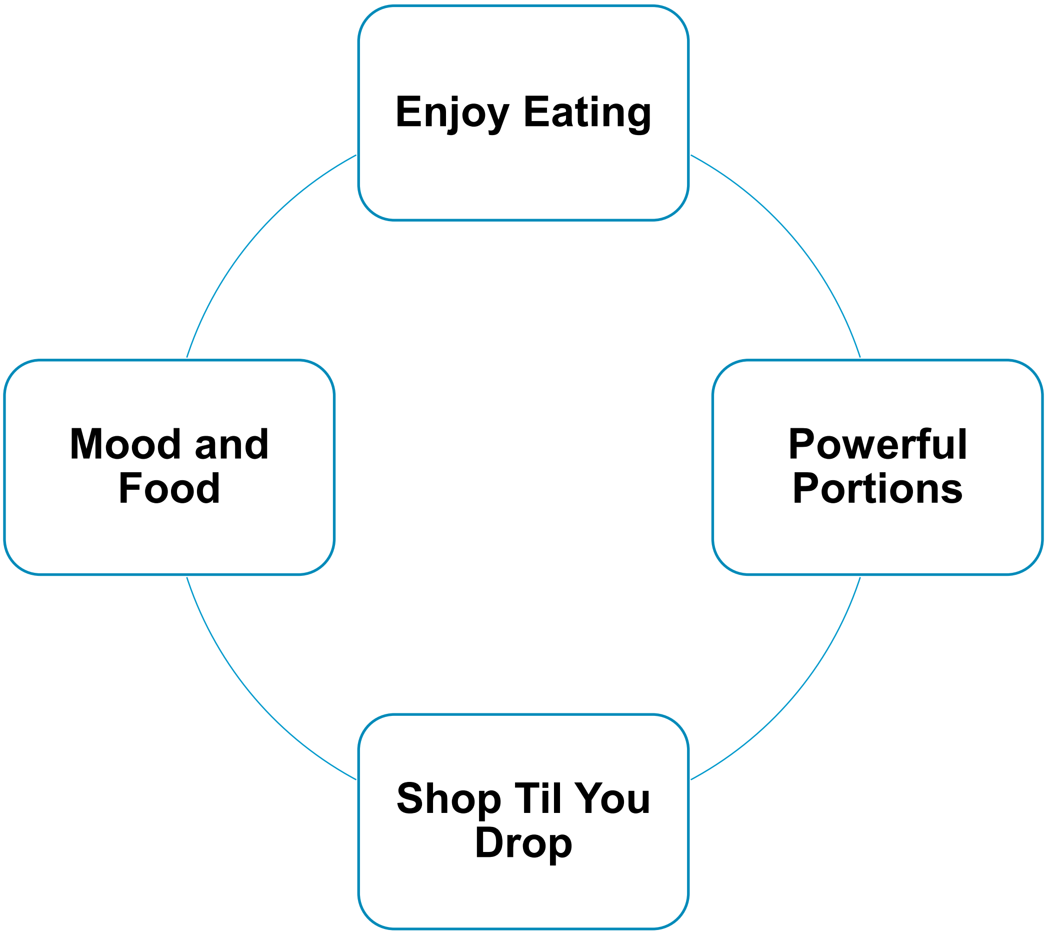

Enjoy Eating

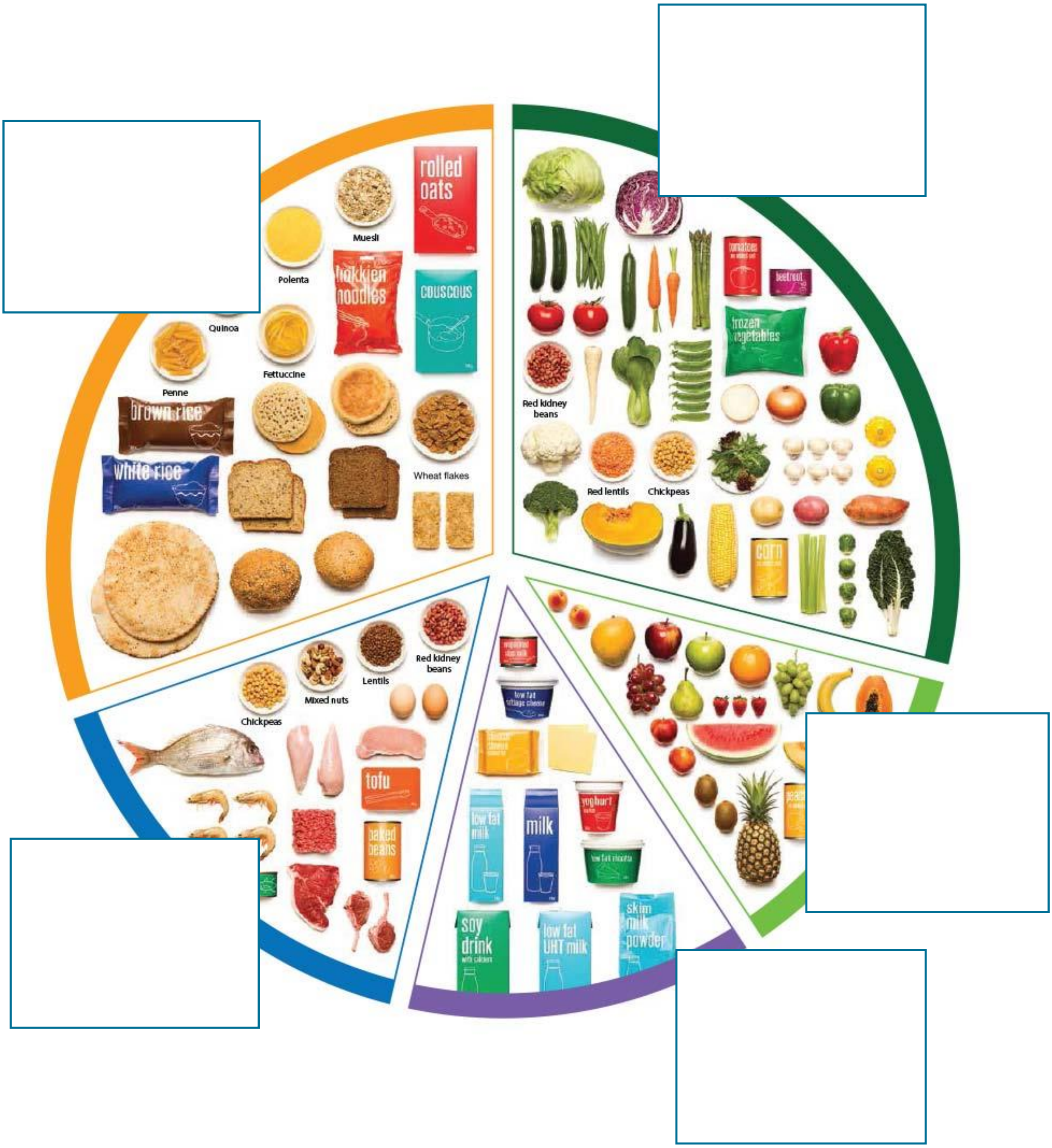

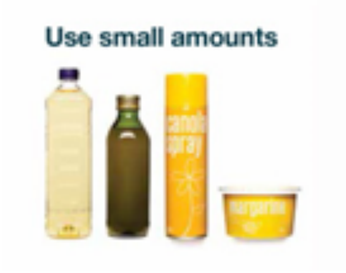

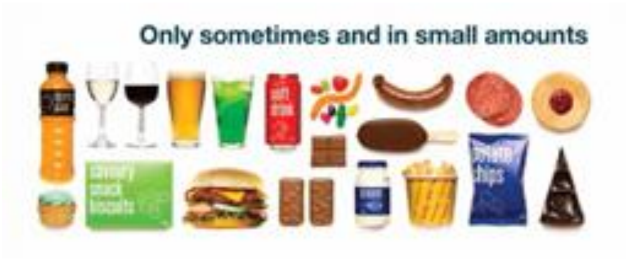

Based on material provided by the National Health and Medical Research Council, The Australian Guide to Healthy Eating

My current eating habits (for completion in the <u>first</u> and <u>last</u> weeks of ATHENA):

Based on the past week please answer the following questions as "yes" or "no", based on what you ate and drank <u>most days</u>:

	**Most days:**	Y	N
1.	I eat 3 meals based on the 5 food groups		
2.	I eat my meals (breakfast, lunch, dinner) at about the same time each day		
3.	I eat regular meals (no skipping or delaying by more than an hour)		
4.	I eat at least 5 serves of vegetables or salad every day		
5.	I eat 1-2 serves of fruit every day		
6.	I have 2 ½ serves of low-fat dairy products every day		
7.	I have 3 serves of lean meat, fish, chicken or legumes every day		
8.	I have mostly whole grain breads and cereals (high fibre)		
9.	My portion sizes (amounts I eat) are the same as the food models shown		
10.	I drink mainly water, unsweetened tea, coffee		
11.	I limit the following foods to once per week		
	• Take-away foods such as pizza, commercial burgers, hot chips		
	• Cakes, muffins, pastries, pies, biscuits and lollies		
	• Salty foods such as processed meats (salami), crisps, salty snacks		

Age (years)	Five Food Group Serves Recommended / Day					Sometimes Foods
	Vegetables, legumes, beans	Fruit	Grain (Cereal) Foods	Lean meat, poultry, fish, eggs, tofu, nuts, seeds, legume	Milk, soy milk, yoghurt, cheese	
19–50	5	2	6	2 ½	2 ½	0–2½
51–70	5	2	4	2	4	0–2½
70+	5	2	3	2	4	0–2

Examples of one serve of food	
Vegetables and legumes/beans	- ½ cup cooked vegetables<br>- 1 cup salad vegetables<br>- ½ medium potato/sweet potato
Fruit 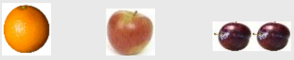	- 1 medium piece (apple, orange, peach)<br>- 2 small pieces (plums, mandarins)
Grain (cereal) foods 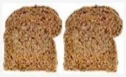	- 1 slice of bread or ½ med bread roll<br>- ½ cup cooked rice or pasta<br>- 2/3 cup cereal flakes or ¼ cup muesli<br>- ½ cup cooked porridge
Lean meats and poultry, fish, eggs, tofu, nuts and seeds and legumes/beans 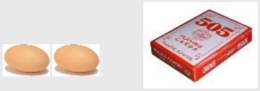	- 65–100g cooked meat, chicken, fish<br>- ½ cup lean mince or 2 slices roast meat<br>- 1 cup cooked/canned beans/legumes<br>- 2 large eggs<br>- 30g nuts, seeds, peanut paste or tahini<br>- 170g tofu
Milk, yoghurt, cheese and alternatives 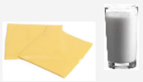	- 1 cup milk or calcium fortified soy milk<br>- 40g cheese (2 slices)<br>- 200g tub yoghurt
Unsaturated oil, margarine, nuts, nut pastes	- 10g poly or monounsaturated spread<br>- 7g polyunsaturated oil, e.g. olive or canola<br>- 10g tree nuts or peanuts, or nut pastes

Sometimes Foods

Some foods do not fit into the five food groups. They are not essential to provide the nutrients the body needs, and some contain too much added fat, salt and sugars. These foods can add to the enjoyment of eating a healthy diet when consumed "sometimes" and in small amounts.

- Biscuits
- Pies
- Cakes
- Pastries
- Chocolate
- Soft drinks
- Alcohol
- Ice cream
- Fried foods
- Confectionary (lollies)

My take home messages

In the next week

I plan to…	I did…

Powerful Portions

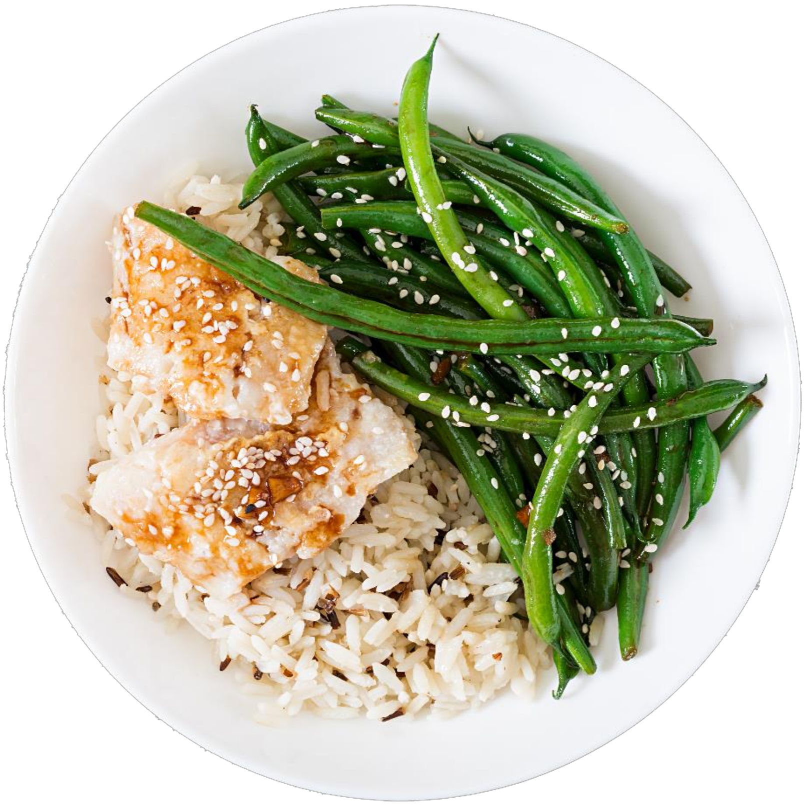

Breakfast Ideas

- High fibre, low sugar breakfast cereal with low fat milk e.g. breakfast biscuits, muesli flakes.
- Natural muesli, low fat yoghurt, berries.
- Toast and egg (poached, boiled or scrambled).
- Tinned fish on toast or English muffin.
- 'Green'/ berry smoothie.
- Baked beans on toast or English muffin.
- Sautéed mushrooms and tomato on toast.
- Raisin toast with a light spread of margarine.
- Add fresh or tinned fruit (in juice) if you are still hungry.

Lunch Ideas

- Sandwich with wholegrain bread; slice of leg ham, lettuce, tomato, cucumber, grated carrot.
- Ham/chicken/tuna and salad flat bread wrap.
- Homemade chicken/meat and vegetable soup with a slice of toast (make extra and freeze).
- Reheat leftovers from the night before.

My lunch ideas (use the following table to help you plan):

2-3 Salad vegetables	1 x 100g Lean meat, poultry etc.	1-2 Carbohydrates
Lettuce<br>Tomato<br>Cucumber<br>Capsicum<br>Grated carrot<br>Sliced salad onion<br>Sliced mushrooms<br>Avocado<br>Tinned beetroot or corn<br>4 bean mix	Lean ham or turkey<br>Cold roast meat e.g. beef or chicken<br>Low fat cheese<br>Tinned tuna, salmon etc.<br>Marinated tofu<br>4 bean mix<br>Steamed fish (try microwaving)<br>Baked Beans<br>Boiled egg	¼ cup rice or potato salad<br>1 slice of wholegrain bread<br>Small bread roll<br>½ small pita bread

- Add a piece of fruit or a small tub of yoghurt if still hungry

Dinner Ideas

Put together your dinner (use the following table to help you plan):

1-2 Vegetables	1 x 100g Lean meat, poultry etc.	1-2 Carbohydrates
Salad vegetables as per lunch<br>Carrots<br>Pumpkin<br>Broccoli<br>Beans<br>Peas<br>Asian Greens<br>Cauliflower<br>Brussels Sprouts<br>Frozen mixed vegetables	Pork<br>Chicken<br>Lamb<br>Beef<br>Fish<br>Egg<br>Legumes<br>Prawns<br>Calamari<br>Tofu or HVP	One small potato<br>One small corn cob<br>½ cup cooked rice<br>½ cup cooked pasta<br>½ cup cooked noodles<br>One slice of wholegrain bread<br>Small bread roll<br>½ cup cooked couscous

In the next week

I plan to…	I did…

Shop Til You Drop

Serving size: judge nutritional value per 100g of food content

Look for items that meet most criteria below:

	Aim for (per 100g)	Avoid (per 100g)
**Total fat**	< 10g	> 10g
**Saturated fat**	< 1.5g	> 3g
**Sugars**	< 15g	> 25g
**Sodium**	<120mg	> 400mg
**Dietary Fibres**	>6g	< 3g

Take home messages

In the next week

I plan to…	I did…

Mood and Food

Our thoughts affect how we feel and guide what we choose to do (such as what we eat and how much we exercise).

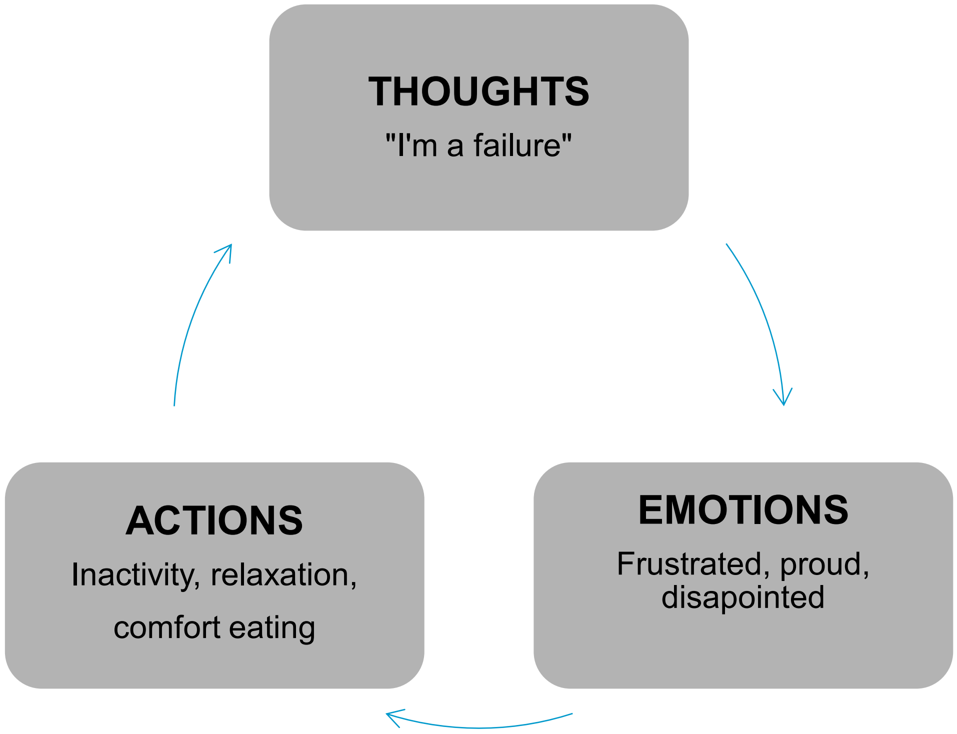

Examples:

**All or Nothing**	"I've failed, I may as well eat the whole block now"
**Permission Giving**	"Today's a right-off so I will start tomorrow"
**Mind Reading**	"My friend will be offended if I don't have a second helping"
**'Should' 'Must'**	"I should be losing weight by now." "I must eat everything on my plate"

Managing Cravings and Emotions

'Stop, breathe, think'

- Be still, calm your mind
- Breathe slowly
- "I can cope"

Environmental focus

- Notice your surroundings – colours/ smells/ objects
- Focus on things external to yourself

Bridging objects

- Look at an object that reminds you of a specific happy memory. E.g. a photograph of a loved one or from a favourite holiday.
- This can help lift your mood

Mental games

- Puzzles or mental games help you to focus on things other than your negative thoughts.
- You can use puzzle books or crosswords or make up your own.

Be involved in an activity or do something fun

- Keeping busy will distract you from negative thoughts
- Planning what you are going to do tomorrow can help keep you motivated

A passive attitude towards thoughts

- Observe your thoughts
- Let your thoughts, images and feelings drift in and out of your mind

Non-mindful Munching

Eating too fast can affect your enjoyment of food and the amount of food you eat. Some helpful techniques for eating slowly:

- Avoid distractions while eating (TV, newspaper etc.)
- Put your cutlery down between bites
- Increase the number of times you chew before swallowing
- Wait 10 min before going for seconds or dessert
- Always eat sitting down at a table

Eating too much can also affect your enjoyment of food. Try:

- Measuring your food with common measurements.
- Serving your food onto a smaller plate.
- Avoid serving from dishes on the table – this encourages seconds
- Let someone else serve your plate

Eating when distracted: certain activities can trigger non-mindful eating; e.g. watching television, talking on the phone. Try:

- Concentrating on eating – i.e. 'mindful eating'
- Breaking the link between eating and certain activities (do I really have to eat while watching a movie?)
- Savouring the flavours of food

Picking at food while cooking/cleaning up can affect your enjoyment of food. To help control the amount you eat, try:

- Not eating while standing.
- Always eat from a plate, not a packet or tub
- Chew sugar-free gum to make it difficult to pick at food

Eating in inappropriate places like the TV room and bedroom can trigger overeating. Try to:

- Restrict your eating to 1-2 rooms (i.e. dining room and living room)

The Dieting Cycle

The dieting cycle is a ‘trap’ that people often get caught up in after many attempts dieting.

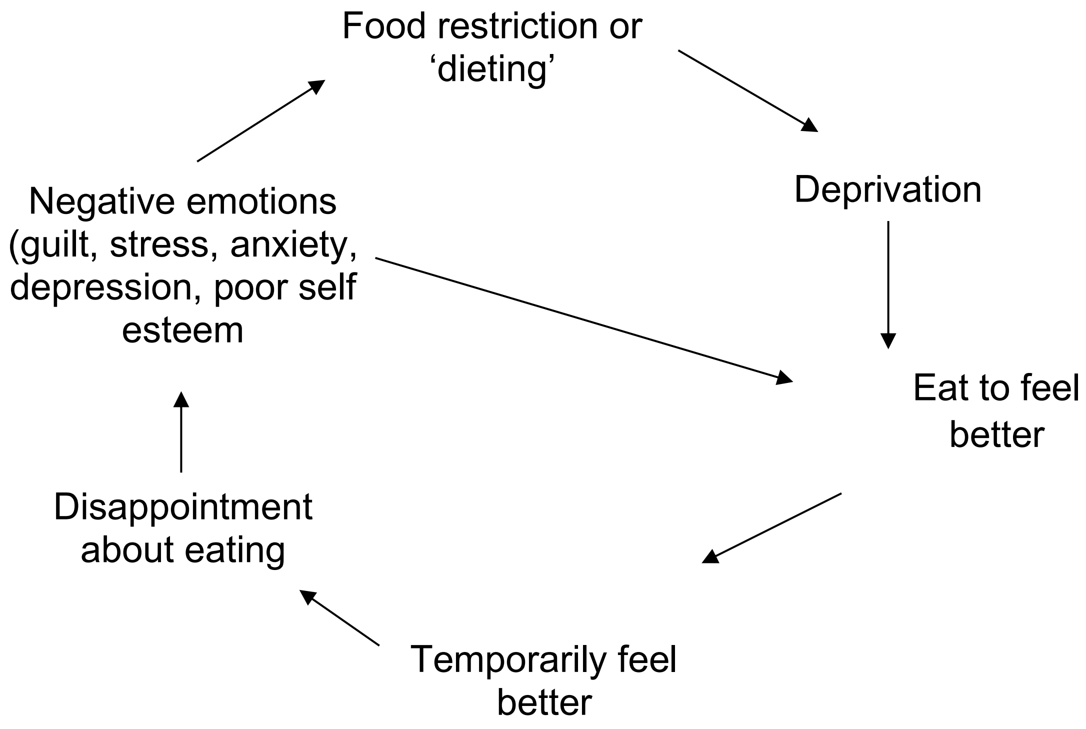

How do I break the cycle?

- Be honest with yourself and your emotions
- Remember that food is neither ‘good’ or ‘bad’
- Avoid depriving yourself completely of food you enjoy
- Enjoy mood boosting activities

Notes

In the next week

I plan to…	I did…

Healthy Sleep Habits

Get a regular routine**:** go to bed and get up at the same time every day.

Sleep when sleepy: only try to sleep when you feel tired.

Get up and try again**:** if you haven't been able to get to sleep for 20 minutes, get up and do something calming or boring and then try again.

Avoid caffeine, nicotine, alcohol**:** 4-6 hours before bed. Stimulants can stop you from getting to sleep, & alcohol from having quality sleep.

Bed is for sleeping**:** try to not use your bed for anything other than sleeping and sex.

Avoid naps**:** during the day to ensure you are tired at night.

Sleep rituals**:** develop rituals to remind your body that it is time to sleep e.g. breathing exercise or relaxing stretches.

Bath time**:** 1-2 hours before bed can increase your body temperature, preparing you for sleep.

Avoid clock-watching**:** checking your clock during the night can wake you up and reinforce negative thoughts.

The right space**:** ensure your bed and bedroom is quiet and comfortable, and you have a plan to block out the morning sun.

Healthy Sleep + Healthy Stress = Happy Hormones

Healthy sleep means getting enough (ideally 7-8 hours/day) and good quality sleep. When we don't get enough and good quality sleep, the following happens to our hormones:

- ↑ **Insulin**: is a fat storing hormone. Increased insulin = increased fat storage.
- ↑ **Ghrelin &** ↓ **Leptin**: Ghrelin is the green light that says "Eat!". Leptin is the red light that tells you to "Stop Eating!". The less you sleep, the hungrier you get, and the more you eat (more than your body's needs).
- ↑ **Cortisol**: is our "fight or flight" hormone. It is caused by stress and lack of sleep and results in burning of not only fat but also muscle. If we lose muscle, we reduce our resting metabolism.
- ↓ **Human Growth Hormone**: helps us burn stored fats and carbohydrates. If we have less, we can't burn fat as well.

Research shows that the less you sleep, the higher likelihood of obesity.

In the next week

I plan to…	I did…

Staying on track

My bladder control and weight management have improved, how can I sustain gains long term?

Once your bladder control and healthy eating have improved, you will need to continue your exercises and healthy habits so that your symptoms do not return.

Your physiotherapist will set you up with a **maintenance program.** Aim to complete this 2-3 x week. Remember pelvic floor exercises, healthy eating habits and good quality sleep need to be a part of your lifestyle long term.

HEALTHY EATING + HEALTHY EXERCISE + HEALTHY SLEEP

=

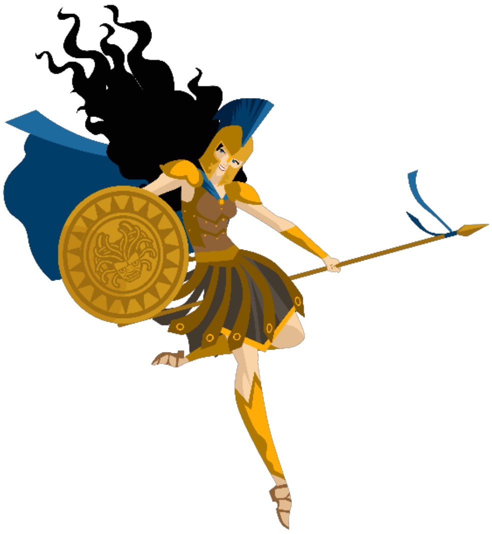

Achieving your inner ATHENA

My General Exercise Training Diary

Week	In the next week I plan to…	I did…
1		
2		
3		
4		
5		
6		
7		
8		
9		
10		
11		
12		

Notes

My Pelvic Muscle (Trampoline) Exercise Training Diary

Week	1	2	3	4	5	6	7	8	9	10	11	12
**Long holds (__seconds) 3 sets x 8-12 reps**												
**Fast flicks 3 sets x 8 – 12 reps**												
**Cough**												
**Sit-stand**												
**Squat**												
**During general exercises**												

Notes

Notes

Notes

For further information visit:

Continence Foundation of Australia	www.continence.org.au
National Continence Helpline	Ph: 1800 33 00 66
Pelvic Floor First	http://www.pelvicfloorfirst.org.au
Australian Dietary Guidelines	www.eatforhealth.gov.au

ATHENA Participant Workbook

Published by the State of Queensland (Queensland Health), 2020

This document is licensed under a Creative Commons Attribution-No Derivatives 4.0 International licence. To view a copy of this licence, visit creativecommons.org/licenses/by-nc-nd/4.0/ © State of Queensland (Queensland Health) 2020

You are free to copy and communicate the work; however, it cannot be shared with others in a modified or adapted form, and you must attribute the State of Queensland (Queensland Health).

For more information contact:
Address: Gold Coast Hospital and Health Service Physiotherapy Department, D Block Ground Floor, 1 Hospital Blvd, QLD 4215. Email: PTLedPelvicHealthClinicGCUH@health.qld.gov.au.
Phone: +61 5687 3027.

Disclaimer:
The content presented in this publication is distributed by the Queensland Government as an information source only. The State of Queensland makes no statements, representations or warranties about the accuracy, completeness or reliability of any information contained in this publication. The State of Queensland disclaims all responsibility and all liability (including without limitation for liability in negligence) for all expenses, losses, damages and costs you might incur as a result of the information being inaccurate or incomplete in any way, and for any reason reliance was placed on such information.
